# To Dr. Batty

**Published:** 1800-08

**Authors:** Richard Dunning

**Affiliations:** Plymouth Dock


### *To Dr.* BATTY.

DEAR SIR,

IF you will inſert the following definition of the Cow-pox, in the next Number of your uſeful Journal, you will add another obligation to the many attentions already ſhewn to

Your obliged humble ſervant,

RICHARD DUNNING.

*Plymouth Dock,*

*July* 7, 1800.

### VACCINA JENNERIANA.

Morbus Vicarius, potiuſve Proceſſus ſuccedaneus, mirifico Variolam cèrte præveniendi, immò (quod vereſimilius ſit) penitùs abolendi fungens munere, neve ullam futuræ Saluti vel minime adverſam Sequelam illaturus.—Cuticulari Vulnuſculo Vaccinitate appoſitâ, Tumor parvus, *localis*, *veſiculoſus*, *carbunculoſus*, inde exortus, Febrem ephemeram plerùmque qùam leviſſimam et omnìno incontagioſam incitans; paucis Papulis, ſubìnde, ſed *perràro*, *nec necessàrie* ſupervenientibus, tertioque ferè die prorſus evaneſcentibus.

### *To Dr.* BRADLEY.

DEAR SIR,

